# Echinacea—A Source of Potent Antivirals for Respiratory Virus Infections

**DOI:** 10.3390/ph4071019

**Published:** 2011-07-13

**Authors:** James Hudson, Selvarani Vimalanathan

**Affiliations:** Department of Pathology & Laboratory Medicine, University of British Columbia/Vancouver, BC V5Z 3J5, Canada; E-Mail: vnathanm@telus.net

**Keywords:** antiviral, *Echinacea*, phytomedicine, respiratory viruses, respiratory infection, anti-inflammatory

## Abstract

Extracts of *Echinacea* species have been used traditionally in North America for the control of symptoms of colds, influenza, and other diseases, and some of them have become very popular as “herbal medicines”. Recent studies have revealed that preparations derived from certain species and plant parts, but not all of them, possess potent antiviral activities, at non-cytotoxic concentrations, particularly against membrane-containing viruses. Thus all strains of human and avian influenza viruses tested (including a Tamiflu-resistant strain), as well as herpes simplex virus, respiratory syncytial virus, and rhinoviruses, were very sensitive to a standardized *Echinacea purpurea* preparation. In mechanistic studies the influenza virus-specific hemagglutinin and neuraminidase were inhibited. In addition some extracts displayed anti-inflammatory activity in virus-infected cells, and numerous other effects on the expression of cellular genes. Multiple components, either discrete compounds or mixtures, appeared to be responsible for the various antiviral activities.

## 1. Introduction

Acute respiratory infections in humans are usually ascribed to one or more of a group of well known viruses, including more than 100 rhinoviruses (“common cold” viruses), influenza viruses A and B, parainfluenza viruses, corona viruses, respiratory syncytial virus, and certain adenoviruses [1-4]. In addition the recent application of more sensitive molecular detection techniques has revealed the presence of other viruses, such as metapneumoviruses and bocaviruses, which might also be involved in the generation of respiratory symptoms. However we do not know if these newly recognized viruses are really pathogenic, or are simply “passengers” that eluded previous diagnostic techniques. Nevertheless various families of viruses, with different structures and replication schemes, and consequently bearing different potential molecular targets, are clearly involved in respiratory symptoms, as indicated in Table 1. Among the possible targets are: (i) the virion itself; (ii) cellular attachment or entry; (iii) one or more of the many stages in virus replication and development, particularly those that involve virus-specific enzymes; (iv) egress of progeny virus from infected cells. However the variety of replication schemes indicated in Table 1 reduces the chances that a single antiviral drug could target many of these viruses [3,5]. In addition, in the majority of respiratory infections specific virus information is lacking; consequently it is difficult to conceive of a single therapeutic agent or regimen that could control the “causative agent”. Nevertheless, in spite of this limitation, considerable time and money has been spent trying to find the “silver bullet” for specific virus “infections”, so far without much success [6].

Another problem with the specific target approach, especially in the case of compounds directed at specific viral genes or their products, is the inevitable emergence of virus resistant mutants and their subsequent spread through the community and environment. The conventional answer to this problem has been the suggestion that two or more antiviral drugs, with distinct molecular targets, be used in combination, notwithstanding the likely increase in undesirable side-effects. A logical alternative approach is the use of a non-toxic agent that has the capacity to inhibit many different respiratory viruses simultaneously, and recent evidence indicates that certain herbal extracts might fulfill this requirement [5,7,8].

## 2. Nature and Causes of Symptoms

“Colds” and “flu” are terms that have been coined to describe a combination of common symptoms, supposedly brought about by the actions of specific viral infections of the upper respiratory tract. These symptoms may include such familiar discomforts as sneezing, stuffy nose, irritation of mucous membranes, excess mucus production, sinusitis, cough, sore throat, malaise and fever, as well as exacerbation of asthma and COPD (chronic obstructive pulmonary disease). In some cases symptoms may spread to include the lower respiratory tract and lungs, and result in bronchitis, bronchiolitis, or pneumonia [1,8,9]. However the symptoms may not be a direct result of virus replication, which in many cases is minimal in airway tissues [10], but rather an indirect consequence of virus-induced inflammatory responses [5,11].

In respiratory infections, whether they start in the nasal passages or oropharynx, or other parts of the airway, the invading virus initially encounters epithelial tissues, composed largely of epithelial cells and occasional dendritic cells and macrophages, which accordingly respond by means of the various antimicrobial strategies that make up the innate immune system, including defense peptides (antimicrobial peptides) and the secretion of various pro-inflammatory cytokines and other mediators of inflammation [12,13]. Other molecules such as kinins are released and are probably responsible for some of the early symptoms. Phagocytic cells and various types of inflammatory cell may then be attracted to the site of infection. In addition the redox balance of the cells may be adversely affected, either by the virus infection itself or as a consequence of the pro-inflammatory response [14].

Since most of the symptoms reflect this common non-specific host response to infecting agents, rather than to the direct cytolytic or cytopathic effects of a specific virus [5,7,8], then a more rational therapeutic approach would be the application of anti-inflammatory agents, especially if the intention of the therapy is to ameliorate symptoms. If a potential safe anti-inflammatory agent also contains multiple antiviral activities, then this would provide a bonus.

The limitations of conventional antiviral therapy and prevention were illustrated in 2002 with the sudden appearance of the SARS (severe acute respiratory syndrome) pandemic. The novel coronavirus responsible for the disease (SARS-CoV) was quickly isolated and its genome sequenced [15]; however no adequate antiviral treatment was deemed to be available at that time.

Several herbal extracts have been shown recently to possess a combination of bioactivities that could be useful in the control of colds, flu, and bronchitis [5,8], and, in retrospect, some of these could have been useful for SARS patients. Among these herbal preparations *Echinacea* extracts have become very popular, although not all of them are necessarily beneficial, as will be discussed.

## 3. Antiviral Properties of *Echinacea*

Herbal preparations of *Echinacea* are usually made in the form of extracts, tinctures, teas, sprays etc. derived from various parts of one or more of three species of *Echinacea: E. purpurea*, *E. angustifolia*, and *E. pallida* (Table 2, and ref. [16]). The other species have received less attention.

However, a problem with commercial *Echinacea* extracts in general (and in common with many other herbal products) is their inadequate characterization and standardization. Consequently different commercial sources, derived from different species and plant parts, and with resulting distinctive chemical compositions, may show different combinations of bio-activities, or in some cases relatively little bioactivity [17]. The result of this is that research studies, and especially clinical studies, have yielded inconsistent results. It is also important to ensure that any antiviral activity detected in a herbal preparation is really significant, that is to say, the extract should be able to inactivate a substantial amount of virus at a practical non-cytotoxic dosage, and therefore the assay techniques should reflect this requirement.

Early reports of antiviral activity of *Echinacea* [18] indicated that several different methanol and aqueous extracts derived from *E. purpurea* could partially protect cultured cells from infection by influenza A virus, herpes simplex virus type 1, or vesicular stomatitis, viruses. This suggested an intracellular inhibition, although the possibility of a virucidal activity was not reported.

Later studies supported the concept of *Echinacea* species as a potential source of antiviral activities. Cheminat *et al.* [19] isolated and characterized a group of caffeoyl derivatives from dried and fresh *E. pallida* plants, and examined two of them, cichoric acid and echinacoside, as well as caffeic acid, a constituent of *E. purpurea*, for activity against the replication of vesicular stomatitis virus (a membrane containing RNA virus) in a mouse cell line. Activity was relatively weak however, except at high and cytotoxic concentrations, and the possibility of virucidal activity was not tested.

Binns *et al.* [20] examined extracts from a variety of different species and plant parts for antiviral activity against herpes simplex virus. Assays were designed to test virucidal activity or viral growth inhibition, and they also incorporated exposure to light in case photosensitizers were involved (these are often found as bioactive constituents of medicinal plants, ref. 21). The results are summarized in Table 3. Many of the extracts showed significant but relatively weak activity, although the hexane root extract of *E. purpurea* and the ethanol inflorescence extract of *E. sanguinea* were more substantial. Pure cichoric acid was also moderately active, and could therefore contribute to the activity of certain extracts.

Vimalanathan *et al.* (Table 3, ref. [22]) evaluated different solvent fractions of *E. purpurea* aerial parts for activity against several viruses, in the presence and absence of light during the reactions. Aqueous extracts were active against herpes simplex virus and influenza virus, but these activities were not dependent on light exposure. In contrast the ethyl acetate fraction of the ethanol extract contained impressive activity against both viruses and which was due to a photosentizer. No activity against rhinovirus was detected. A polysaccharide-enriched fraction was also tested and found to contain only a relatively weak activity. Data are summarized in the form of MICs (minimum inhibitory concentrations) in Table 4.

Similar studies were carried out to compare root extracts from *E. purpurea*, *E. angustifolia* and *E. pallida* (23). The aqueous fraction of *E. purpurea* roots, which was almost devoid of caffeic acid derivatives and alkylamides, showed impressive activity against HSV and influenza virus (Table 4). The *E. angustifolia* root extract contained moderate activity against all three viruses (HSV, influenza, and rhinovirus) in the ethyl acetate fraction, but no activity in the aqueous extracts. In contrast, *E. pallida* root extracts were devoid of antiviral activity.

However, in a more recent study, a series of aqueous and ethanol extracts of *E. pallida* aerial parts showed significant virucidal activity against HSV-1 and HSV-2 [24], and some of the extracts also appeared to inhibit virus replication within infected cells. The different extracts had distinct chemical profiles, as expected, but the authors concluded that combinations of components, rather than individual compounds, were responsible for these different activities. In recent tests we found that an ethanol extract of *E. angustifolia* aerial parts was as active against HSV as a corresponding *E. purpurea* extract (unpublished observations), thus indicating that there may be similar antiviral compounds in aerial part extracts of all three common *Echinacea* species (*E. purpurea, E. angustifolia*, and *E. pallida*). It is also clear that several different compounds contribute to the overall antiviral activity of a given extract, although many other extracts are devoid of activity.

## 4. The Need for Standard Extracts

The presence of multiple antiviral activities among different extracts and fractions suggests that many kinds of *Echinacea* preparation, such as tinctures, sprays, tablets, teas, etc. could be beneficial in the treatment of colds and flu, although not all preparations are likely to be effective. In fact a recent study on 10 commercial preparations highlighted the variability of antiviral activity between different preparations, although lot-to-lot variation was less evident [17]. In general ethanol based extracts had greater antiviral activity than aqueous extracts; but it was not possible to identify a specific component responsible for the activity. Furthermore there was no correlation between antiviral activity and anti-inflammatory activity.

Recent detailed studies with the standardized preparation Echinaforce^®^ (comprising ethanol extracts of *E. purpurea*, 95% aerial parts plus 5% roots; abbreviated EF) showed that this preparation was very active as a virucidal agent against viruses with membranes, as indicated in Table 2. In addition to HSV-1 and respiratory syncytial virus, all tested human and avian strains of influenza A virus, as well as influenza B virus, were susceptible [25,26]. In addition rhinovirus was also equally susceptible at the relatively high concentrations of Echinaforce^®^ recommended for oral consumption (1:10 dilution, equivalent to 1.6 mg/mL dry weight/volume). Under these conditions more than 10^5^ infectious viruses could be killed within 5 min.

In contrast Echinaforce^®^ was found to be less effective against intracellular virus [25]. Consequently virus already present within a cell could be refractory to the inhibitory effect of Echinaforce^®^, but virus particles shed into the extracellular fluids would be vulnerable [25,26]. Therefore the actions of the Echinaforce^®^ would be manifest during initial contact with the virus, i.e. at the inception of infection, and also during transmission of virus from infected cells.

Additional experiments showed that continuous passage of influenza A virus in cell cultures in the presence of Echinaforce^®^ did not result in the emergence of resistant strains, whereas passage of the virus through successive cultures in the presence of Tamiflu rapidly generated Tamiflu-resistance. Furthermore Tamiflu-resistant virus remained fully susceptible to Echinaforce^®^ [26]. Therefore continuous usage of Echinaforce^®^ in the population would be less likely to generate resistant strains of virus than Tamiflu or other anti-influenza compounds currently in the market. Recent studies have illustrated the relative ease with which resistant strains of influenza virus can arise [6,27]. Furthermore Echinaforce^®^ could also be useful as an accessory treatment for individuals undergoing anti-influenza therapy with agents such as Tamiflu.

It was shown by hemagglutination assays that this extract (EF) inhibited the receptor binding activity of influenza A viruses, over a range of EF concentrations including that recommended for oral consumption, suggesting that EF interfered with viral entry into the cells, thus effectively rendering the virus non-infectious [26]. EF also inhibited neuraminidase activity *in vitro* (unpublished results), suggesting that the active compounds could block influenza virus entry and spread by acting on at least two virion targets. However, the susceptibility of other viruses, which do not rely on HA or NA functions, to Echinaforce^®^ indicates that additional molecular targets must be accessible.

## 5. Mucin Secretion

Most sufferers of colds and other respiratory problems would agree that secretion of excessive mucus is one of the more annoying symptoms, and accordingly many pharmaceuticals have been designed to relieve this feature of a cold or flu, usually with the accompaniment of undesirable side effects. Rhinoviruses induced the secretion of excess MUC5A, the dominant respiratory mucin, in bronchial epithelial cells in culture, and in cultured airway tissues, and Echinaforce^®^ reversed this secretion in both systems [28], suggesting that this could be an additional benefit of *Echinacea* treatment. This result was supported by histochemical examination of cultured airway tissues, which revealed the conspicuous presence of muco-polysaccharide-filled goblet cells resulting from rhinovirus infection, whereas EF treated and infected tissues appeared normal [28].

## 6. Effects on Virus-Infected Cells

A series of studies by Sharma *et al.* focused on the application of *E. purpurea* extracts (including Echinaforce^®^) to epithelial cells and tissues infected by viruses [25,29-31]. In rhinovirus infected human bronchial and lung epithelial cell lines the virus could stimulate the secretion of more than 30 different cytokines, including the pro-inflammatory IL-1, IL-6, IL-8, and TNFα, which are known to be collectively involved in many of the symptoms common to colds and flu, such as sneezing, fever, sore throat, nasal discharges and inflammation in various respiratory tissues. Certain *Echinacea* preparations were able to completely or partly reverse this stimulation [25,29-31]. In studies with Echinaforce^®^, it was shown that EF could be added before or after virus infection, with similar success, and also the results were not affected by virus dose or the time of exposure to EF [29].

Other viruses, including HSV-1, influenza A virus, adenovirus type 3 and 11, and respiratory syncytial virus, stimulated the secretion of pro-inflammatory cytokines, and in each case the stimulation was reversed by EF (Table 5, and ref. [25]). However only live infectious viruses were able to do this, for infection by equivalent doses of ultraviolet-inactivated viruses failed to elicit the responses. This suggests that the virus may have to enter the cells and undergo some degree of gene expression in order to stimulate the cytokine expression or secretion. It is also interesting that viruses such as adenoviruses, which are not vulnerable to direct attack by *Echinacea*, but could nevertheless stimulate cytokine secretion, were still susceptible to cytokine reversal.

In an attempt to correlate immune modulatory effects with specific classes of *Echinacea* components, various chemically characterized extracts and fractions, derived from three common species of *Echinacea*, were evaluated for their possible inhibitory effects on the secretion of pro-inflammatory cytokines IL-6 and IL-8 (CXCL-8) by human bronchial epithelial cells infected with rhinovirus type 14. All of the *E. purpurea* fractions, comprising aqueous or ethanol extracts of roots, leaves and stems, but to a lesser degree flowers, strongly inhibited the secretion of both cytokines [32]. These results suggest that different compounds, or combinations, were responsible for antiviral and anti-inflammatory effects.

## 7. Effects on Cellular Gene Expression

Several studies have reported the effects of *Echinacea* preparations on cellular gene expression, mostly in uninfected cultured cells relevant to the immune system, although it is not feasible to compare the studies because of the different cellular systems and because the *Echinacea* preparations were different.

Randolph *et al.* [33] described changes in levels of expression (in terms of mRNAs and proteins) of several cytokine genes in human blood samples taken at different times after treatment with a commercial blended *Echinacea* product, and Brovelli *et al.* [34] found that the expression of several cytokine genes in cultured human monocytes was influenced by the nature of the *Echinacea* preparation (stage of development and plant part used), presumably a reflection of their different chemical compositions. Altamirano-Dimas *et al.* [35] analyzed gene expression in human bronchial cells by means of DNA microarrays, following treatment by one of two *E. purpurea* preparations, a polysaccharide rich aqueous extract and an alkylamide-rich ethanol extract, with or without infection by rhinovirus type 14. Both extracts influenced the expression of many genes, including cytokine genes, although the pattern of expression was different for the two extracts. In addition the virus induced numerous changes, mostly increases in expression, and the extracts tended to decrease (i.e. restore to normal levels) these expression levels. Further analysis of the effects revealed that some of the changes in cytokine expression were interconnected through a specific transcription factor, C/EBPb (CAAT/enhancer-binding protein b). Since Sharma *et al.* [31] had shown that numerous transcription factors were affected by *E. purpurea* extract in this same cell-virus system, it is tempting to conclude that many gene expression effects of *Echinacea* extracts could be due to changes in expression or activation status of multiple transcription factors. This in turn could be brought about by interaction with surface receptors or intracellular modulators.

However in an extension of these analyses Altamirano-Dimas *et al.* [36], using cytokine arrays for > 50 cytokines and chemokines, observed that changes in cellular gene expression of some cytokines were not apparently reflected in corresponding protein expression. Thus the interactions between a particular *Echinacea* extract and target cells, infected or otherwise, are complex and may involve different levels in the signaling pathway network. Nevertheless the important conclusion of these analyses is that rhinovirus induction of numerous cytokines and their secretion can be modulated by an appropriate *Echinacea purpurea* extract.

Wang *et al.* [37] described the effects of a butanol fraction, derived from aerial parts of *E. purpurea*, on gene expression of immune-related molecules in human dendritic cells, which are part of the adaptive immune response, in contrast to the studies described above, which focused on the innate immune response. Many dendritic cell genes were affected, either up regulated or down-regulated. These studies did not include infected cells, but clearly showed the multiple gene effects of this *Echinacea* preparation.

Benson *et al.* [38] studied the effects of extracts of *E. purpurea*, an aqueous extract of roots and an ethanol extract of aerial parts, on selected immune-related proteins in murine dendritic cells, and found a variety of significant responses, reinforcing the concept of multiple consequences of *Echinacea* exposure of immunologically important cells.

## 8. Mechanisms

The results reviewed above indicate that certain *Echinacea* extracts contain multiple bio-activities which collectively inactivate and/or inhibit many viruses at different levels. Influenza viruses present at least two molecular targets, the hemagglutinin and neuraminidase proteins; but other viruses evidently contain additional virion targets. There are also intracellular mechanisms at play, which include a widespread reversal of virus-induced pro-inflammatory cytokine secretion, mediated through one or more signaling pathways. All of these events occur at non-cytotoxic concentrations.

The lack of correlation between antiviral activities and the presence of the known marker compounds for *Echinacea* extracts, i.e. caffeic acid derivatives, alkylamides and certain types of polysaccharides, prompted us to search for evidence of alternative bioactive compounds. We found recently that the antiviral activity in ethanol extracts of *E. purpurea* aerial parts was able to bind to the polymeric matrix polyvinyl-polypyrrolidone (PVPP) and could subsequently be eluted and recovered (Hudson and Vimalanathan, unpublished results). Recent studies on PVPP have shown its effectiveness in selectively removing tannins and other polyphenols from plant extracts, by a variety of chemical interactions [39]. Thus the antiviral components of *E. purpurea* aerial parts could be, or could include, polyphenols. Anti-inflammatory activities could be due partly to alkylamides or other constituents.

In view of the numerous effects of *Echinacea* extracts on gene expression, additional consequences can be anticipated, and some of these could also supplement the beneficial effects of *Echinacea* in counteracting virus infection. If the active compounds can be isolated and characterized, then further analyses could lead to improvements in the efficacy of the extracts, and in addition provide further evidence to substantiate some of the medical claims made for *Echinacea.*

## 9. Conclusions

Studies on *Echinacea* extracts have shown that some of them, but not all, possess multiple beneficial actions in the treatment of viral respiratory infections: (1) a direct virucidal activity against several respiratory viruses; (2) reversal of the pro-inflammatory response of epithelial cells and tissues to different viruses; (3) reduction in the excessive secretion of mucin by airway cells and tissues; (4) lack of cytotoxic effects or disruption of tissue integrity by *Echinacea* in airway cell cultures or tissues, at practical antiviral concentrations; (5) additional potentially positive effects on cellular gene expression. A combination of these beneficial activities could reduce the amount of prevailing viable virus, and their transmission, and also lead to amelioration of the virus-induced symptoms.

## Figures and Tables

**Table 1:** Respiratory viruses and their potential targets.

**Virus**	**Relevant properties**	**Potential targets**	**Susceptible to *Echinacea*(±)^1^**
Influenza viruses A & B (FluV A/B) (Orthomyxoviridae)	Segmented ssRNA genome + membrane	Hemagglutinin, neuraminidase (others ?)	+
Respiratory syncytial virus (RSV) (Paramyxoviridae)	ssRNA + membrane	Membrane components	+
Parainfluenza viruses (PI 1-4), (Paramyxoviridae)	ssRNA + membrane	Membrane components	?
Metapneumoviruses (hMPV) (Paramyxoviridae)	ssRNA + membrane	Membrane components	?
Coronaviruses (HCoV, SARS CoV) (Coronaviridae)	ssRNA + membrane	Membrane components	+
Rhinoviruses, coxsackieviruses, (Picornaviridae)	ssRNA, no membrane	Capsid proteins, replication	+
Adenoviruses (Adenoviridae)	dsDNA, no membrane	Capsid proteins, replication	-
Herpes viruses HSV-1/2 (Herpesviridae)	dsDNA + membrane	Membrane components virus replication	+
Bocavirus (HBoV) (Parvoviridae)	ssDNA, no membrane	Capsid proteins	?

1 details and references in text (Section 3).

**Table 2:** Taxonomy of *Echinacea* species.

**Botanical name**	**Usual common name**	**Popular name used in scientific literature**
*Echinacea purpurea* (L.) Moench	Purple coneflower	*E. purpurea*
*Echinacea pallida* var *angustifolia* (DC.) Cronq.	Narrow leaf coneflower	*E. angustifolia*
*Echinacea pallida* var *pallida* (Nutt.) Cronq.	Pale purple coneflower	*E. pallida*
*Echinacea atrorubens* var *atrorubrens*	Topeka purple coneflower	*E. atrorubens*
*Echinacea laevigata* (Boynton and Beadle) Blake	Smooth coneflower	*E. laevigata*
*Echinacea atrorubens* var *paradoxa* (J. B. S. Norton) Britt.	Yellow coneflower	*E. paradoxa*
*Echinacea pallida* (Nutt.) var *sanguinea* (Nutt.) Gandhi & R. D. Thomas	Sanguine purple coneflower	*E. sanguinea*
*Echinacea pallida* (Nuttall) Nuttall var *simulata* (McGregor)	Wavyleaf purple coneflower	*E. simulata*
*Echinacea pallida* var *tennesseensis* (Beadle) Small	Tennessee coneflower	*E. tennesseensis*

**Table 3:** Antiviral activities of *Echinacea* species.

*Echinacea* **sp. and plant part**	**Susceptible viruses**	**References**
*E. purpurea* aerial parts	Influenza virus A (human and avian); influenza B; HSV-1 and -2; coronavirus; respiratory syncytial virus; rhinoviruses	[18,19,22,26]
*E. purpurea* roots	Influenza A, HSV-1	[23]
*E. angustifolia* aerial parts	Influenza A, HSV-1, rhinovirus	[22]
*E. angustifolia* roots	HSV-1	[23]
*E. pallida*, aerial parts & roots	HSV-1 and -2	[24]
*E.sanguinea*, inflorescence	HSV-1, influenza A	[20]
Other species	Weak or no activity	[20]

**Table 4:** Antiviral MICs (minimum inhibitory concentration, μg/mL).

**Plant source**	**Type of extract**	**Antiviral activity MIC_100_ (μg/mL)**
*E.purpurea* aerial	aqueous	HSV 9.8
		FluV 19.6
	ethanol	HSV 3.5
		FluV 5.8
*E. purpurea* root	aqueous	HSV 1.4
		FluV 2.4
	ethanol	NS (> 100)
*E. angustifolia* root	aqueous	NS (> 100)
	ethanol	HSV 13
		FluV 22
		RV 72

Data taken from refs. [22] and [23]. HSV, herpes simplex virus type 1; FluV, influenza virus H3N2; RV, rhinovirus 14; NS, not significant.

**Table 5:** Cytokines/chemokines induced by viruses (+) and reversed by Echinaforce^®^.

**Cytokine**	**RV**	**FluV**	**RSV**	**Ad 3**
IL-1a	+	+	+	+
IL-5				+
IL-6	+	+	+	+
IL8 (CXCL-8)	+	+	+	+
TNFα	+	+	+	+
GROα	+	+	+	
CCL-3			+	+
CCL-4			+	+

RV, rhinovirus; FluV, influenza virus; RSV, respiratory syncytial virus; Ad 3, adenovirus type 3.
